# Comparing specific capacitance in rice husk-derived activated carbon through phosphoric acid and potassium hydroxide activation order variations

**DOI:** 10.1038/s41598-023-49675-0

**Published:** 2024-01-17

**Authors:** Nasser A. M. Barakat, Mohamed S. Mahmoud, Hager M. Moustafa

**Affiliations:** 1 Faculty of Engineering, Chemical Engineering Department, Minia University, El-Minia, 61516 Egypt; 2 Department of Engineering, University of Technology and Applied Sciences, Suhar, 311 Oman

**Keywords:** Energy science and technology, Materials science

## Abstract

This manuscript investigates the influence of the chemical activation step order and process parameters on the specific capacitance of activated carbon derived from rice husk. The chemical activation was performed either before or after the carbonization step, using phosphoric acid (H_3_PO_4_) and potassium hydroxide (KOH) as activating agents. For activation before carbonization, the carbonization process was conducted at various temperatures (600, 750, 850, and 1050 °C). On the other hand, for activation after carbonization, the effect of the volume of the chemical agent solution was studied, with 0, 6, 18, 21, 24, and 30 mL/g of phosphoric acid and 0, 18, 30, 45, 60, and 90 mL/g of 3.0 M KOH solution. The results revealed that in the case of chemical activation before carbonization, the optimum temperature for maximizing specific capacitance was determined to be 900 °C. Conversely, in the case of chemical activation after carbonization, the optimal volumes of the chemical agent solutions were found to be 30 mL/g for phosphoric acid (H_3_PO_4_) and 21 mL/g for potassium hydroxide (KOH). Moreover, it was observed that utilizing phosphoric acid treatment before the carbonization step leads to an 21% increase in specific capacitance, attributed to the retention of inorganic compounds, particularly silica (SiO_2_). Conversely, when rice husks were treated with KOH after the carbonization step, the specific capacitance was found to be doubled compared to treatment with KOH prior to the carbonization step due to embedding of SiO_2_ and KHCO_3_ inorganic constituents. This study provides valuable insights into the optimization of the chemical activation step order and process parameters for enhanced specific capacitance in rice husk-derived activated carbon. These findings contribute to the development of high-performance supercapacitors using rice husk as a sustainable and cost-effective precursor material.

## Introduction

Supercapacitors have emerged as a promising energy storage technology, offering high power density, fast charging and discharging rates, and long cycling life. They have the potential to revolutionize various applications, including portable electronics, electric vehicles, and renewable energy systems^1,2^. The key component of a supercapacitor is the electrode material, which directly influences its energy storage performance^3,4^.

Rice husk, an agricultural byproduct abundantly available worldwide, poses a significant environmental challenge due to its disposal. However, rice husk can be effectively utilized as a precursor for the production of activated carbon (ACs), a commonly used electrode material in supercapacitors. In general, the conversion of renewable biomass to ACs is considered as more worthwhile when considering production costs and energy/environmental effects^5,6^. Until now, ACs have been synthesized from various renewable biomasses, such as rice husk^7,8^, cellulose^9–11^, and lignin^12,13^. Around 571 million tons of rice are produced annually in the world, yielding around 140 million tons of useless rice husk. As a result, transforming renewable rice husk into electrode materials is thought to be a cost-effective and long-term solution^14,15^. This approach offers a sustainable and cost-effective solution for the utilization of rice husk, reducing waste and providing a valuable resource for energy storage applications^16^.

Rice husk is characterized by its unusually high ash content, with silica (SiO_2_) accounting for nearly 20% of its total weight^17^. Additionally, it contains metallic impurities in low concentrations. The high ash content in rice husks poses challenges in the preparation of activated carbon, as it hinders the development of pores, resulting in activated carbon with reduced mechanical strength and adsorption capacity compared to activated carbon derived from low-ash lignocellulosic materials^18^. Furthermore, during the chemical activation process, the activating agent reacts with the silica in the ash, leading to a decreased activating agent/carbon ratio and lower surface area compared to activated carbon derived from low-ash precursors^19^.

To overcome the limitations imposed by the high ash content of rice husks, chemical activation for the initial rice husk involving the use of acids or bases have been employed. The beforehand treatments effectively reduce the ash content of rice husks by as much as 92-98wt%, depending on the used agent's concentration, pretreatment ratio, soaking time, process temperature, and pH^20,21^. Consequently, the preliminary chemical treatment increases the BET surface area, widens the pore size distribution, and creates a new pore structure in the resulting activated carbon^22^. Acid-treatment primarily removes the remaining metallic impurities, which would otherwise obstruct pore development^23^. On the other hand, base-treatment predominantly removes silica by forming soluble potassium silicate (Na_2_SiO_3_), which can be easily washed away^21^.

In the context of utilizing activated carbon derived from rice husks, it is important to consider the presence of metallic impurities and their influence on different applications. While metallic impurities in the rice husk are generally undesired when using activated carbon for adsorption processes, they can offer advantages in the realm of supercapacitors. In adsorption applications, metallic impurities can hinder the pore development and reduce the adsorption capacity of activated carbon^24^. Consequently, efforts are made to remove these impurities through chemical treatmnet processes^18,25,26^. However, in the case of supercapacitors, the presence of metallic impurities can be beneficial. These compounds can contribute to the pseudocapacitance of the activated carbon, leading to improved energy storage performance^27,28^. Therefore, the selection of chemical treatment techniques and the control of metallic impurity content depend on the specific application of the activated carbon, highlighting the importance of understanding the advantages and disadvantages of metallic impurities in different contexts.

Graphitization and carbonization are processes that involve the transformation of carbon-containing materials, particularly biomaterials, into carbon structures. While both processes result in the formation of carbon, there are distinct differences between them. Carbonization is a thermal decomposition process that involves heating carbonaceous materials in the absence of air or in a low-oxygen environment. This process leads to the elimination of non-carbon elements, such as hydrogen and oxygen, from the original material, leaving behind a carbon-rich residue. Carbonization is commonly applied to various biomaterials, including wood, plants, and agricultural residues, to produce materials like charcoal and activated carbon. During carbonization, the temperature plays a crucial role in determining the final properties of the resulting carbon material. Lower temperatures typically lead to the formation of amorphous carbon, while higher temperatures can result in the development of more ordered carbon structures.

Graphitization is a more specific process that occurs at higher temperatures, typically above 2500 °C, and involves the rearrangement of carbon atoms into a highly ordered graphite structure. This process is characterized by the growth of graphene layers, forming a crystalline structure with well-defined planes. While carbonization can be a step in the graphitization process, not all carbonization processes lead to graphitization. Graphitization is often associated with synthetic carbon materials, such as those derived from petroleum-based precursors or certain carbonization processes applied to biomaterials.

The sequence of chemical treatment and carbonization steps in the preparation of activated carbon from rice husks significantly impacts its quality. Researchers have reported that the sequence of carbonization-activation-leaching yields activated carbon with superior textural parameters compared to the sequence of carbonization-leaching-activation^29,30^. However, it should be noted that the used chemical compounds may lead to lower carbon yields since the lignin, the main constituent for char formation, is removed after base-treatment. Liginin can be removed from rice husk by several chemicals including potassium carbonate and sodium hydroxide^31,32^. Moreover, in case of sodium hydroxide, the removal efficiency can be enhanced by the addition of other compounds. For instance, Bazirgan et. al. optimized the conditions for alkaline peroxide pretreatment of rice husks and found that the most effective conditions were 5.29% NaOH**,** 1% H_2_O_2_**,** and 20 °C. Under these conditions, a solid yield of 50.89% was achieved, considering the initial concentration of different components in rice husk (28.21% lignin, 16.5% ash, and 5.01% extractives)^33^. Another study focused on maximum removal of impurities and found that under the conditions of 8% NaOH**,** 1% H_2_O_2_**,** and 20 °C, 71.78% lignin removal, 88.47% silica removal, and 50.89% solid yield were achieved^34^. Addition of ethanol has been also proposed to improve the alkaline removal of ethanol from the rice husk^35^. Accordingly, it is safe to claim that the proposed alkaline treatment in this study will lead to removing the lignin partially.

A careful control of the chemical treatment process is necessary to prevent compromising the yield and quality attributes of the resulting activated carbon. It is noteworthy mentioning that the sequence of treatment steps (i.e. the chemical treatment and the carbonization processes) has been intesively studied in case of utilizing the produced activated carbon in the adsorption process. On the other hand, based to our best knowledege, there is a lack of information about the influence of the sequence of the activated carbon production steps when it is exploited in the supercapacitors.

Although there are several chemicals that have been utilized in the chemical route preparation of rice husk-based rice husk, phosphoric acid and potassium hydroxide have drawn the maximum attention^36,37^. Potassium hydroxide (KOH) has been extensively employed as an activation agent, even reaching commercial utilization. For example, Amoco Corporation utilized KOH to convert aromatic precursors like coal and petroleum coke into high surface area carbons with approximately 3000 m^2^/g. Kansai Coke and Chemicals Co. obtained a license to employ this method on a pilot plant scale after its commercialization in the 1980s. The substantial surface area of the activated carbon is attributed to its predominantly microporous structure, resulting in a high total pore volume of 2.0–2.6 mL/g^38,39^.

This manuscript focuses on investigating the influence of the chemical activation step order on the specific capacitance of activated carbon derived from rice husk. Specifically, we examine the effect of performing chemical activation before or after the carbonization step. Additionally, we explore the impact of process parameters such as carbonization temperature in the case of activation before carbonization and the volume of the chemical agent solution in the case of activation after carbonization.

By optimizing the activation step order and process parameters, we aim to enhance the specific capacitance of rice husk-derived activated carbon, thereby improving its suitability for supercapacitor applications. The findings of this study contribute to the utilization of rice husk as a valuable precursor material for sustainable and high-performance energy storage devices.

## Materials and experimental procedures

### Materials

All chemicals were of analytical grade and used without further purification. The chemicals used were orthophosphoric acid (H_3_PO_4_, 85%, SDFCL, Germany), sulfuric acid (H_2_SO_4_, 98%, Scharlau, Spain), and potassium hydroxide pellets (KOH, Oxford Lab Fine Chem LLP, India). Rice husk samples were collected from a local farm in Gharbia Governorate, Egypt. It was cut into small pieces, washed, and dried at 110 °C for 24 h. The dried rice husk has a moisture content lower than 5%. The dried rice husk has a moisture content lower than 5%. The moisture content of rice husk was measured by using a primary method, based on weight measurements (oven method) before and after drying. First, the sample was left in the oven until the weight remained constant. Then, the moisture content for the one day-dried samples were calculated accordingly. The samples were ground and sieved to collect particles of a suitable size range.

### Activated carbon preparation

#### Activation then carbonization route

An activator is used to activate rice husk to get the activated carbon powder. 15 mL of H_3_PO_4_ or 15 mL of KOH (3 M) are used as activators with 0.3 g of dried rice husk for all experiments. Accoringly, the used chemicals were utilized in the form 45 ml/g_carbon_. The impregnation step was performed by reflux at 100 °C for 24 h. Then, the rice husk activated samples were washed with hot water to remove residual acid or base. The neutral samples were then dried at 110 °C for 24 h. Later on, the samples were carbonized at different temperatures (600, 750, 900, and 1050 °C) in a furnace for 2 h. After carbonization, the obtained materials were cooled down to room temperature in a desiccator to be ready for an electrochemical tests.

#### Carbonization then activation route

The rice husk material, after being thoroughly cleaned and dried, underwent carbonization in a furnace at 600 °C for a duration of 2 h. Subsequently, the resulting rice husk char was allowed to cool to room temperature within a desiccator. The activation process was carried out using reflux mode, employing solutions of KOH and H_3_PO_4_ in varying quantities. A consistent amount of 0.3 g of carbonized rice husk was used for all experiments. To ensure effective interaction between the activation agent and the rice husk, the suspension of rice husk char and the activation agent underwent reflux for 3 h. Different volumes of H_3_PO_4_ (stock: 6, 18, 21, 24, and 30 mL/g_carbon_) and KOH (3M concentration: 18, 30, 45, 60, and 90 mL/g_carbon_) were employed for each individual investigation. Subsequently, the treated samples were thoroughly washed with hot water to remove any residual acid or base, with the washing process continuing until the pH approached 7. The neutralized rice husk activated carbon samples were then dried at 110 °C for 24 h and subsequently cooled n a desiccator.

### Preparation of electrodes and electrochemical measurements

The supercapacitor study was conducted using a three-electrode cell configuration. In order to prepare the electrode, 2 mg of sample was dispersed by ultrasonically mixing with 400 μl of isopropyl alcohol and 5% Nafion 117 solution. Drop casting was employed to apply the produced ink on a glassy carbon electrode (GCE), which served as the working electrode. The counter electrode was made of platinum, and the reference electrode was made of Ag/AgCl. A 1.0 M aqueous H_2_SO_4_ electrolyte was used for the electrochemical studies.

### Characterization

The FT-IR analysis is done using Nicolet Avatar 370 spectrometer. It was used for the investigation of the surface functional groups of the prepared activated carbon samples. The samples were mixed with KBr of spectroscopic grade and made into pellets at a pressure of about 1 MPa. The pellets were about 10 mm in diameter and 1 mm in thickness. The samples were scanned in the spectral range of 4000–400 cm^−1^. X-ray diffraction experiments were performed using a Bruker D8 diffractometer with Cu-Ka radiation at 40 kV and 40 mA; λ = 1.5406 Å for 2θ values from 10 to 80° along with a low angle. Other experimental conditions were 1/2° divergence slits and a 5-s residence time at each step. The reflected beam intensity was measured in counts.

## Results and discussion

### Phase identification and chemical composition

**Figure 1:**
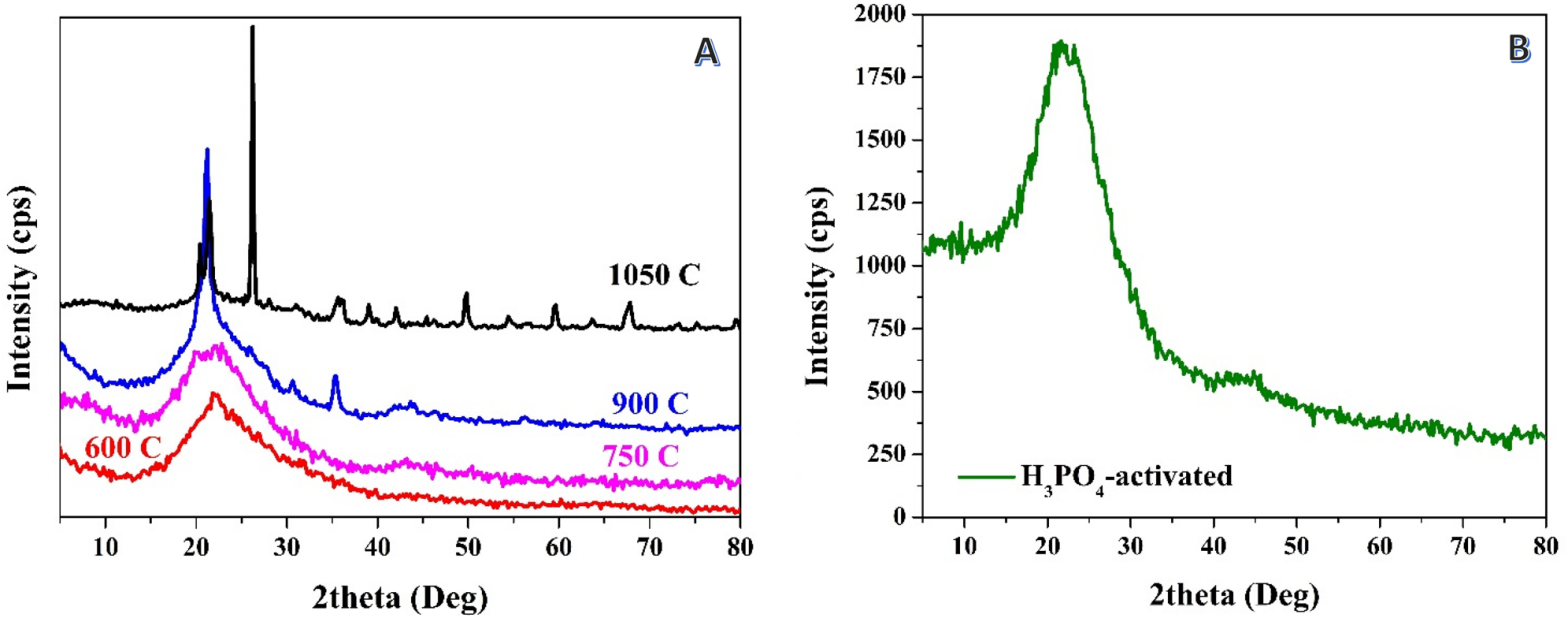
XRD patterns for the prepared activated carbon from rice husk using phosphoric acid in chemical activation step: (**A**) the acid was used before carbonization (at several temperatures), (**B**) the acid was used after carbonization at 600 °C.

The XRD results of the samples treated with phosphoric acid prior to the carbonization process revealed interesting findings; Fig. 1A. At low carbonization temperatures of 600 and 750 °C, no peaks indicating the presence of inorganic compounds were observed. However, as the carbonization temperature increased to 900 °C, XRD analysis showed the appearance of peaks associated with silica. Typically, appearance of peaks at two theta values of 20.51°, 26.18°, 41.48°, 45.05°, 49.5° and 58.93° corresponding to (100), (101), (200), (201), (112) and (211) crystal plans confirm formation of silica based on The International Centre for Diffraction Data – ICDD card# 11-0252. This suggests that some silica residues were retained or formed during the carbonization process at this high temperature, despite the prior treatment with phosphoric acid. The presence of silica at this temperature could be attributed to the high thermal stability of silicon-containing organic compounds, which were not decomposed at the relativley low temperatures; 600 and 750 °C. Observably, the XRD pattern corresponding to the sample graphitized at 1050 °C exhibited sharper peaks representing silica. This suggests that the carbonization process at higher temperatures enhanced the crystallinity of the retained silica, resulting in more distinct diffraction peaks. The increased sharpness of the silica peaks indicates a higher degree of ordering in the silica structure. These findings highlight the influence of carbonization temperature on formation of silica in the activated carbon derived from rice husk.

It is noteworthy mentioning that utilizing the chemical activation post-to-the carbonization process has been investigated by several reseachers. It was concluded that the carbonization temperature does not have a strong impact on the final product composition^40,41^. Acid-treatment can eliminate metallic impurities due to the high dissolution affinity of the metals in acid^24^. According to Liou et al.^26^, around 84% of the metallic impurities could be extracted by refluxing the graphitized rice husk with 3 N HCl at 100 °C for 1 h. As can be concluded from Fig. 1B, utilizing of phosphoric acid after the carbonization process resulted in dissolution the metallic impurties from the produced char which in consistient with the other reports^42,43^. Accordingly, it can be claimed that, to maintain some metallic compounds in the rice husk-originated activated carbon, it is recommended to utlize the acid before the carbonization step.

**Figure 2:**
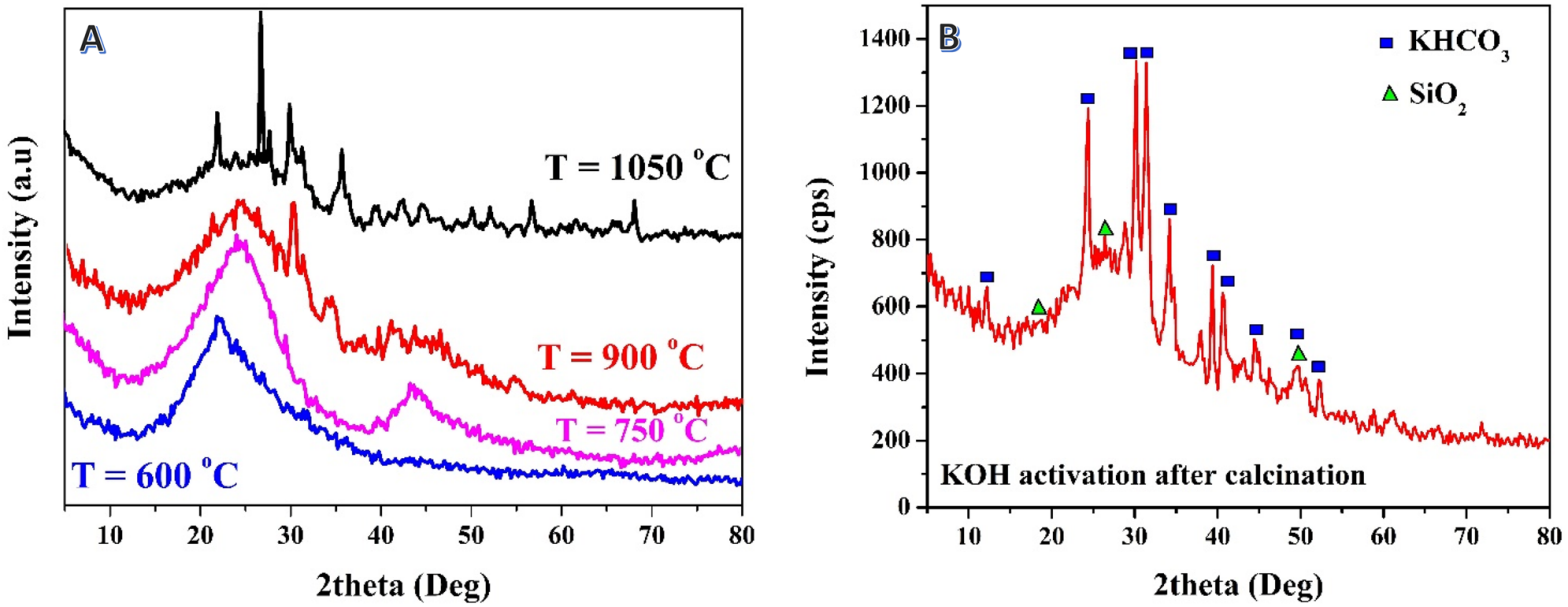
XRD patterns for the prepared activated carbon from rice husk using potassium hydroxide in the chemical activation step: (**A**) the base was used before carbonization (at several temperatures), (**B**) the base was used after carbonization at 600 °C.

The XRD results of the samples treated with potassium hydroxide (KOH) prior to the carbonization process are depicted in Fig. 2. As shown, like the acid-treatment (Fig. 1A), at low carbonization temperature (600 °C), no peaks indicating the presence of inorganic compounds were observed. However, in contrast to acid, a broad peak, associated with silica, appeared at 2θ value of ~ 45° at a calcination temperature of 750 °C. Moreover, the other silica representing peaks appeared clearly at 900 °C and became sharper at 1050 °C. The variation in silica detection between the two treatments can be attributed to the differences in their mechanisms of action. Phosphoric acid likely reacts with silica, either removing it or causing its transformation, even at lower temperatures. On the other hand, the KOH treatment might not have the same level of interaction with silica.

Alkaline activation is commonly employed after the carbonization stage and has demonstrated favorable performance. Previous studies have indicated that the reaction between KOH and graphitized rice husk can lead to the formation of new chemical compounds^44–46^. This hypothesis was confirmed in the current investigation, as depicted in Fig. 2B. The XRD pattern of the sample treated with KOH reveals the presence of KHCO_3_ compound in the resulting activated carbon. The emergence of distinct diffraction peaks at two theta values of 12.0°, 24.2°, 28.8°, 30.0°, 31.2°, and 34.1°, corresponding to crystal planes (200), (400), (201), (-311), and (211), respectively, indicates the formation of Kalicinite (#12-0292). The infiltration and intercalation of KOH into the carbon layers are crucial factors to consider when analyzing the impact of potassium in the activation process^47^. However, in contrast to previous studies that employed KOH as an activation agent at high treatment temperatures^48^, the current study conducted the activation process using a reflux system. Hence, the formation of KHCO_3_ is reasonable. Specifically, when KOH is employed, it undergoes dehydration (at 400 °C) during the conventional thermal activation, resulting in the separation of carbon layers. Consequently, a portion of the potassium forms alkalides such as -OK groups and K_2_CO_3_^49^. The presence of silica in the sample is also evident from the XRD analysis. Potassium hydroxide reacts with silicon dioxide to generate potassium metasilicate, potassium metatetrasilicate, and water. This reaction occurs at temperatures ranging from 900 to 1000 °C. However, the alkali reflux treatment demonstrated significant efficacy in removing silica, as indicated by the minimal amount of SiO_2_ detected in the resulting activated carbon. It is known that the acidic groups in the prepared activated carbon can be neutralized by various bases^50^. Thus, the presence of the prominent intensity peak of oxygenated activated carbon after the activation process using a KOH solution could be attributed to the neutralization of numerous oxygenated groups during the activation step. This hypothesis is supported by the study of Wenwen Zhao et al., who investigated the neutralization of graphene oxide using different chemicals^51^. The study concluded that neutralizing the oxygenated groups leads to a decrease in the intensity of the graphene oxide peak in the XRD pattern.

The surface area of a capacitor electrode material plays a significant role in determining its specific capacitance. In the context of electrochemical capacitors like supercapacitors, specific capacitance measures the charge storage capacity per unit mass or area of the electrode material. A larger surface area enhances specific capacitance by providing more sites for ions in the electrolyte to adsorb and desorb at the electrode–electrolyte interface, ultimately increasing the overall charge storage capacity. Furthermore, a larger surface area facilitates improved charge transfer and reduces the current density at specific points on the electrode, which helps maintain higher capacitance levels without local ion concentration or polarization. To measure the surface area of electrode materials, the Brunauer–Emmett–Teller (BET) method is commonly employed, utilizing gas adsorption isotherms, particularly with nitrogen gas, to determine the specific surface area in square meters per gram (m^2^/g), thereby quantifying the material's accessibility for ion adsorption and its direct impact on specific capacitance. Table 1 summarizes the surface area of the prepared samples:

**Table 1:** Surface area (m^2^/g) for the prepared samples.

Acid treatment		Alkali treatment	
Before carbonization	After carbonization	Before carbonization	After carbonization
118.8	204.5	113.6	156.1

The results indicate that, in both cases, the post-treatment resulted in increasing the surface area of the prepared activated carbon. Moreover, in the case of acid treatment, the post-treatment leads to a distinct increase in the surface area.

### Function groups identification

The FTIR results obtained from the post-carbonization acid treatment reveal important information about the functional groups present in the activated carbon samples. In the FTIR spectra, several peaks were observed, indicating the presence of specific functional groups resulting from the chemical interactions between the activated carbon and the acid treatment.

**Figure 3:**
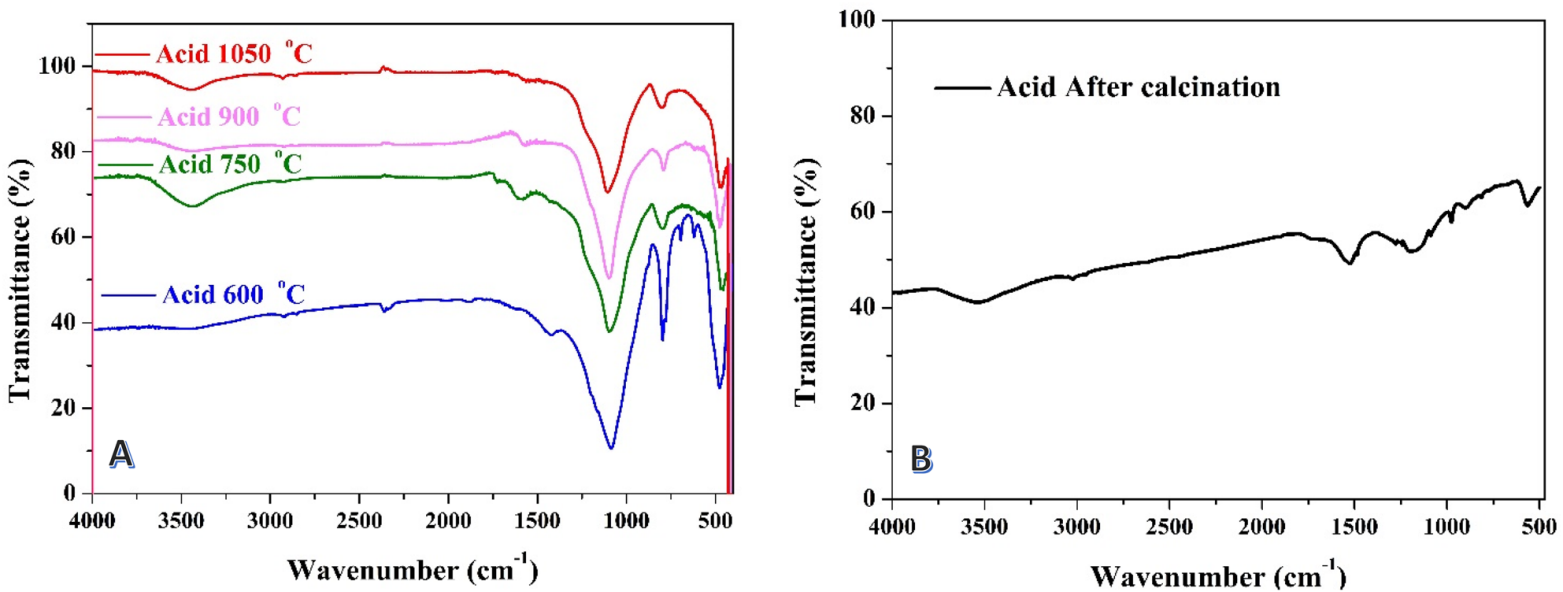
FTIR spectra for the prepared activated carbon from rice husk using phosphoric acid in chemical activation step: (**A**) the acid was used before carbonization (at several temperatures), (**B**) the acid was used after carbonization at 600 °C.

As shown in Fig. 3A, when the acid treatment was conducted before carbonization, a relatively strong peak appeared at 795 cm^−1^, along with a high-intensity peak at 1080 cm^−1^. The appearance of peaks at 795 and 1080 cm^−1^ suggests the presence of active functional groups, possibly including aliphatic C-H bending and C–O stretching vibrations, respectively. Carbonized carbon that undergoes chemical treatment is anticipated to exhibit high ions electrosorption due to the low adsorption affinity of saturated carbon-hydrogen bonds. Stretching of Si–O–Si bonds is observed at 1108 cm^−1^, which coincides with the formation of phosphates resulting from phosphoric acid activation. Additionally, stretching of Si–H bonds is detected at 795 cm^−1^. Consequently, the presence of these bands is specifically associated with phosphorous and phospho-carbonaceous compounds^23,52^. Additionally, a small broad peak was observed at 3450 cm^−1^ in the spectrum which can be attributed to OH bending.

On the other hand, when the acid was exploited after the carbonization process, other peaks were observed at 1180 cm^−1^ and 1531 cm^−1^ in the FTIR spectrum (Fig. 3B) indicating the presence of additional functional groups resulting from the acid treatment. These peaks could be associated with carboxylic acid (C=O stretch) and aromatic C=C bonds, respectively^53,54^.

The detection of these functional groups highlights the significance of acid treatment and carbonization temperature in modifying the surface chemistry of the activated carbon. By controlling the choice of the acid treatment and the carbonization temperature, it is possible to tailor the functional groups present on the surface of the activated carbon, thus influencing its potential applications. The identified functional groups, including carboxylic acid, aromatic C=C bonds, aliphatic C–H bending, and C–O stretching vibrations, can play a crucial role in the electrochemical performance of the activated carbon. These functional groups may contribute to enhanced capacitance, improved charge storage, and modified surface properties, which are desirable characteristics for applications such as supercapacitors^55–57^.

**Figure 4:**
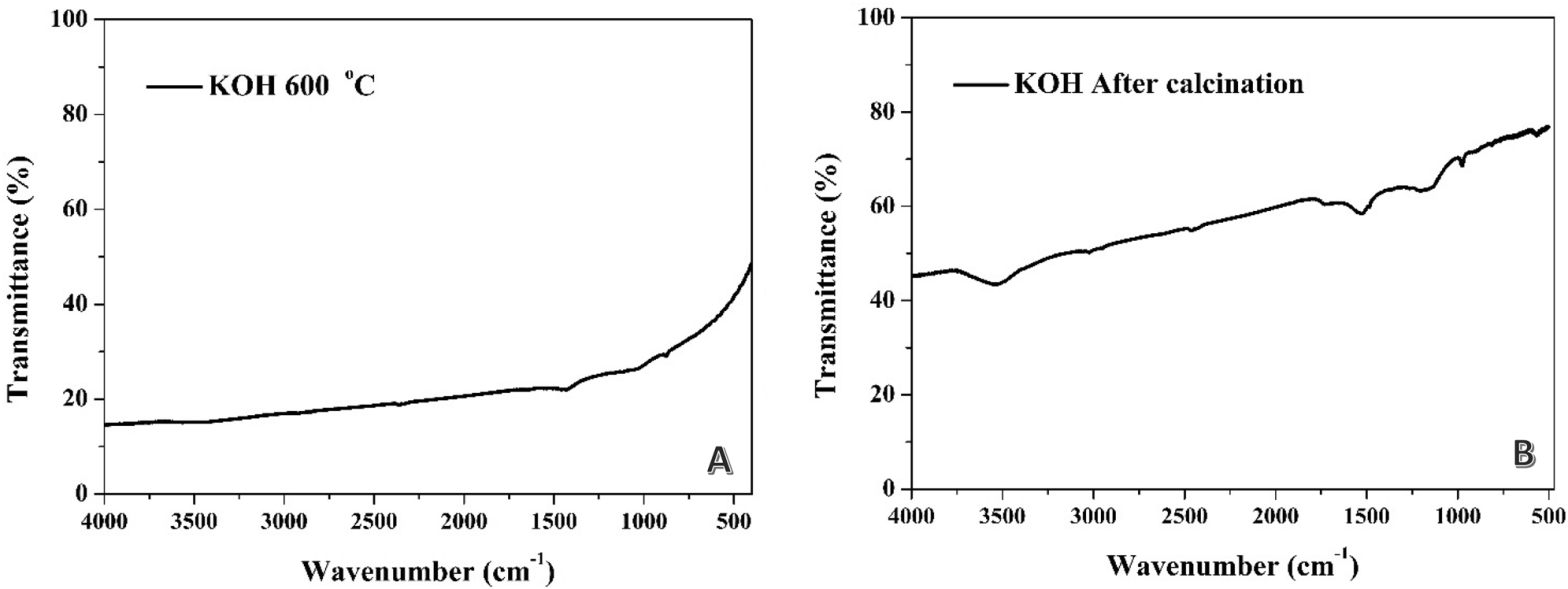
FTIR spectra for the prepared activated carbon from rice husk using potassium hydroxide in the chemical activation step: (**A**) the base was used before carbonization (at several temperatures), (**B**) the base was used after carbonization.

The FTIR results obtained from the alkali treatment prior to calcination reveal important insights into the functional groups present in the activated carbon samples. In the FTIR spectrum (Fig. 4A), only a very small peak was observed at around 1417 cm^−1^ after carbonization at 600 °C. However, for the remaining formulations at 750, 900, and 1050 °C, no peaks could be detected in the spectra (data are not shown). The absence of significant peaks in the FTIR spectra suggests a minimal presence of specific functional groups resulting from the alkali treatment and subsequent carbonization at higher temperatures. This indicates that the alkali treatment followed by carbonization at elevated temperatures effectively removes or transforms the functional groups present in the precursor material, leading to a more graphitic carbon structure with fewer detectable functional groups. The small peak observed at 1417 cm^−1^ may correspond to carboxylic acid (C=O stretch), although its intensity is significantly lower compared to the other treatments.

The FTIR results obtained from the post-carbonization alkali treatment (Fig. 4B) reveal specific functional groups present in the activated carbon samples. The broad peak observed at 1166 cm^−1^ may correspond to specific functional groups, potentially related to C–O or C–N bonds. This peak's broad nature suggests the presence of multiple vibration modes or the overlapping of different functional groups. The small peak observed at 1530 cm^−1^ indicates the presence of another functional group in the activated carbon. This peak's position suggests the involvement of aromatic C=C stretching vibrations. It is worth noting that the intensity of this peak is relatively small compared to the broad peak at 1166 cm^−1^, indicating a lower abundance or weaker vibration of this functional group. At 3528 cm^−1^, a small broad peak appeared in the FTIR spectrum, indicating the presence of functional groups associated with O–H stretching vibrations. This peak suggests the existence of hydroxyl (–OH) groups or water molecules absorbed on the surface of the activated carbon. The presence of hydroxyl groups can significantly affect the surface chemistry and reactivity of the activated carbon, potentially influencing its adsorption properties and interactions with other substances.

It is important to note that the absence of detectable peaks in the FTIR spectra does not necessarily imply the complete absence of functional groups. It is possible that the functional groups present in the activated carbon samples after alkali treatment and carbonization are present at such low concentrations or exhibit weak vibrational modes that they cannot be detected by FTIR spectroscopy. It is noteworthy mentioning that, scanning electron microscope (SEM) analysis did not show a difference in morphology between the prepared activated carbon as shown in Fig. S1 in the supporting information. Moreover, EDS analyses of the samples carbonized at 900 °C are shown in Fig. S2 in the supporting information. It is evident that the sample treated with KOH or H_3_PO_4_ displayed a lower atomic percentage of carbon than the chemically inactivated sample. This finding can be attributed to releasing carbonaceous compounds during the chemical treatment process.

### Electrochemical measurements

#### Post-carbonization acid treatment

**Figure 5:**
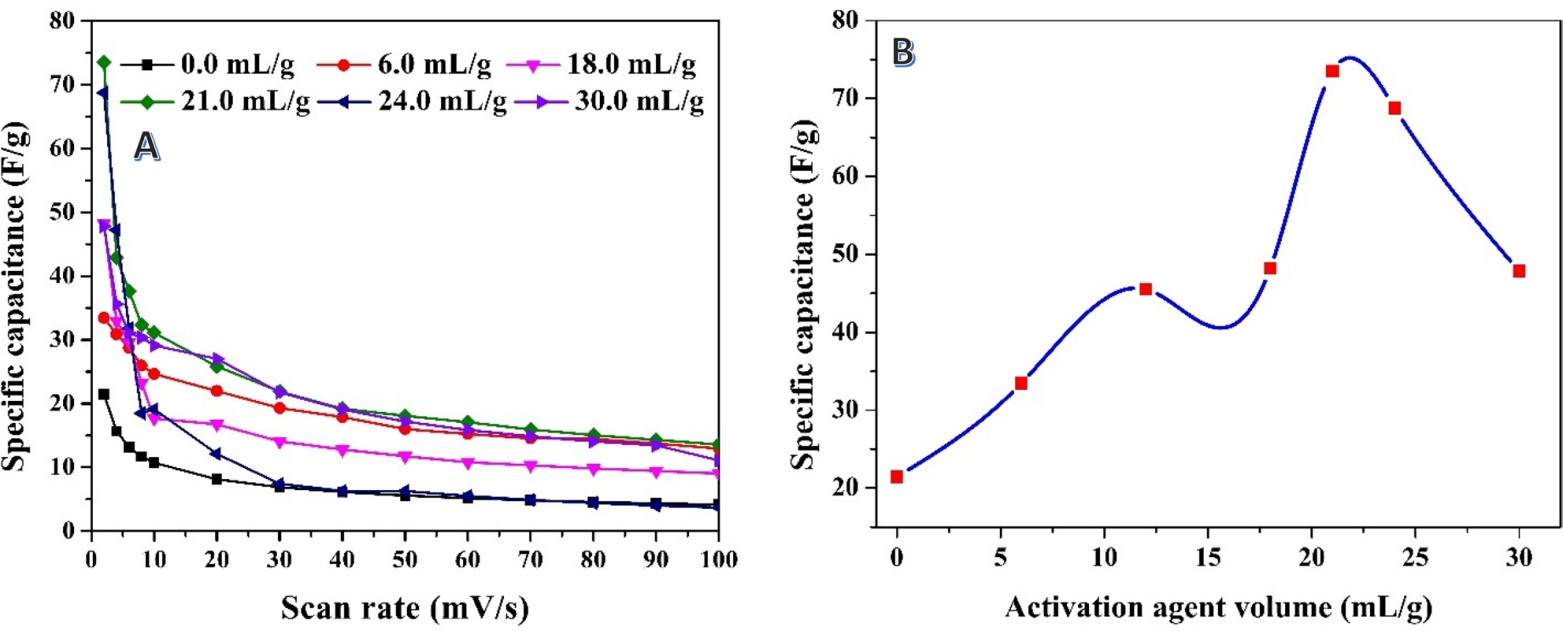
Relationships between scan rate and the specific capacitance at different volumes of the used phosphoric acid in activation of previously graphitized rice husk at 600 °C (**A**), and effect of the used acid volume on the specific capacitance at 2 mV/s scan rate (**B**).

The results obtained from the post-carbonization acid treatment (Fig. 5) demonstrate the influence of phosphoric acid volume on the specific capacitance of the activated carbon. Different volumes of phosphoric acid were utilized in the experiment, ranging from 0 to 10 mL/g for 0.3 g rice husk char. As shown in Fig. 5A, the estimation of specific capacitance at different scan rates revealed a consistent trend: the specific capacitance decreases as the scan rate increases. This behavior is expected and can be attributed to the limited charge/discharge time available at higher scan rates, resulting in reduced ion diffusion and lower capacitance values. The results, in Fig. 5B, also highlight the significant impact of the acid volume on the specific capacitance of the produced carbon. Among the acid volumes tested, it was observed that the specific capacitance exhibited distinct variations. Specifically, the maximum specific capacitance value of 73 F/g was achieved when utilizing 21 mL/g of phosphoric acid at a scan rate of 2 mV/s, which is applied in many previous works^58–62^. The observed dependence of specific capacitance on acid volume suggests that the acid treatment plays a crucial role in the development of the activated carbon's electrochemical properties. The acid likely facilitates the formation of desirable porous structures, enhances the surface area, and promotes the creation of active sites for charge storage. The specific capacitance is, therefore, influenced by the extent of interaction between the acid and the carbon matrix.

#### Prior-to-carbonization acid treatment

**Figure 6:**
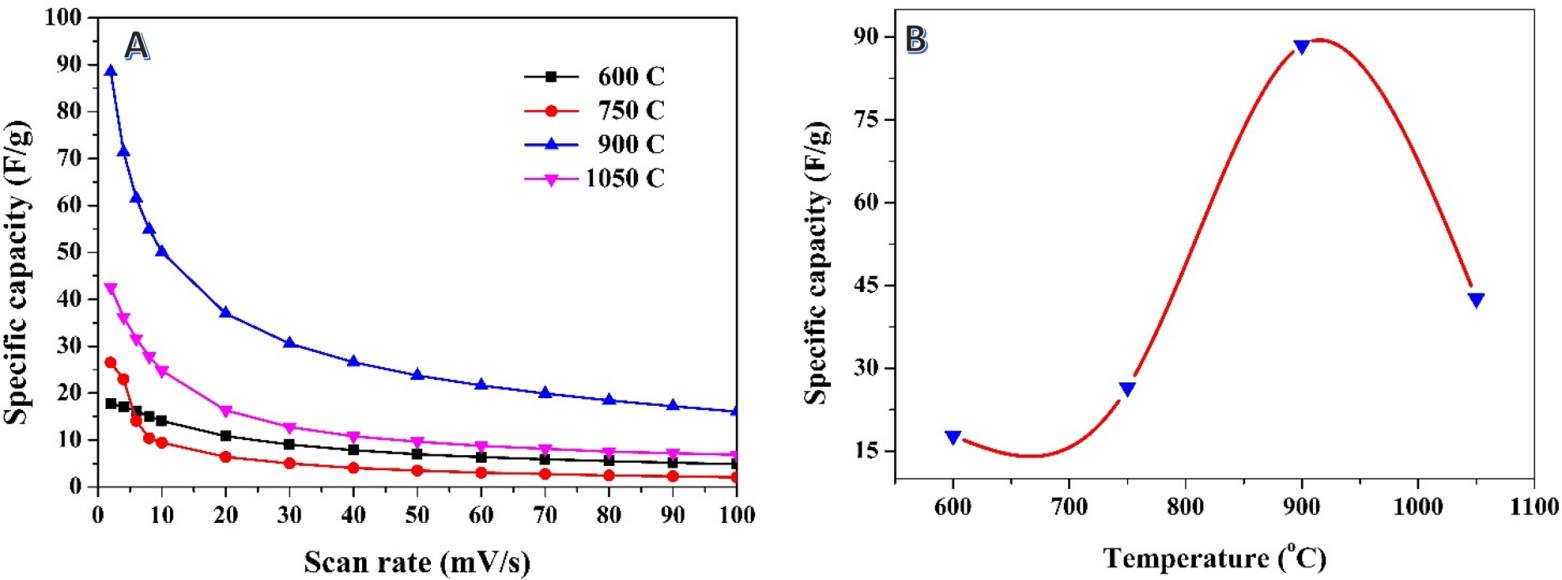
Relationship between scan rate and the specific capacitance at different carbonization temperatures for previously soaked rice husk in phosphoric acid (**A**), and effect of the carbonization temperature on the specific capacitance at 2 mV/s scan rate (**B**).

The results obtained from studying the impact of carbonization temperature in the acid treatment conducted before the carbonization process provide valuable insights into the specific capacitance of the produced activated carbon; Fig. 6. Different temperatures, namely 600, 750, 900, and 1050 °C, were investigated to understand how the carbonization temperature influences the electrochemical performance; Fig. 6A. Consistent with the findings from the post-carbonization chemical treatment samples, the results indicate that the specific capacitance decreases as the scan rate increases at all carbonization temperatures. This trend is expected as higher scan rates limit the charge/discharge time and reduce ion diffusion, resulting in lower specific capacitance values.

Interestingly, the investigation also reveals that the carbonization temperature plays a crucial role in determining the specific capacitance of the activated carbon. Among the temperatures studied, it was observed that the best carbonization temperature for achieving high specific capacitance is 900 °C as can be seen in Fig. 6B. At this temperature, the estimated specific capacitance reaches 88.5 F/g at a scan rate of 2 mV/s, representing an 21% increase compared to the first route (referring to the acid treatment after carbonization).

The improved specific capacitance at 900 °C suggests that this temperature is optimal for the development of desirable structural and electrochemical properties in the activated carbon. It likely promotes the formation of a well-defined graphitic structure, enhances the surface area, and creates favorable conditions for efficient charge storage. These findings underscore the significance of optimizing the carbonization temperature to maximize the specific capacitance of the activated carbon. The relationship between carbonization temperature and specific capacitance is crucial to tailor the electrochemical performance of supercapacitors. Further investigations can delve into the underlying mechanisms that govern the effects of carbonization temperature on the structural and electrochemical characteristics of the activated carbon, providing deeper insights into the optimization strategies for enhanced supercapacitor performance.

#### Post-carbonization alkali treatment

Like phosphoric acid activation, the CV results for KOH activation do not exhibit any redox peaks (data are not shown), indicating the absence of pseudo-capacitance in the analyzed samples. Pseudocapacitive materials have electrochemical properties that are neither bulk Faradaic nor completely capacitive. Between electric double layers capacitors (EDLCs) and redox-based materials, which, according to the traditional definition, rely primarily on the surface Faradaic electron transfer to metal centers that is made possible by the intercalation or adsorption of charge-compensating ions, pseudocapacitive materials stand in the middle^63^. Since pseudocapacitor is not electrostatic in origin but shares similarities with EDLC in terms of cyclic voltammetry (CV) morphologies, it may be seen as a supplementary version of EDLC. The "pseudo" prefix is therefore used to set it apart from EDLC. Nanomaterials are tiny in size and have a huge surface area, making it difficult to discern between the "surface" and the "bulk" in this situation. As a result, some materials for faradaic electrodes that generally exhibit significant redox reactions in bulk have behaviors similar to those of pseudocapacitive materials when their size is decreased to the nanoscale, which is indicative of the removal of the redox peaks in CVs^64^. In this respect, the ion diffusion length has been drastically reduced and the so-called "bulk redox reaction" has essentially converted to the "surface redox reaction." So, following nanosizing, several redox-based materials either show drastically increased redox kinetics (rapid charging rate with high-capacity retention) or pseudocapacitive signs in CVs profiles^64–67^. Accordingly, the nanomaterial exhibiting the pseudocapacitive signature show CVs profile free from the redox peaks, although these peaks clearly appear in the bulk size CVs profile.

For instance, in case of LiCoO_2_, redox CV peaks are typically observed upon the intercalation/ de-intercalation of lithium ion in the host structure. However, it is interesting to find that there are situations where the common battery characteristics of LiCoO_2_ disappear when the material size reaches a critical value. Specifically, when the LiCoO_2_ dimension is reduced from 17 to 6 nm, and, in particular, 6 nm LiCoO_2_ exhibits a redox peaks-free CVs profile as “pseudocapacitive” behavior with faradaic process^64^. Dunn et al.^68^ provided their ideas that those like LiCoO_2_ can be categorized as materials with “extrinsic pseudocapacitance”; and for extrinsic pseudocapacitive materials, in the bulk phase, they behave as battery materials, but after the size reduction, pseudocapacitive behavior emerges. The introduced material in this study does not obey the aforementioned discussion as it is not in the nanoscale and there are not redox peaks in the CVs plot as shown in Fig. S3 in the supporting information. Accordingly, it is safe to claim that the synthesized activated carbons do not behave pesudocapactive.

**Figure 7:**
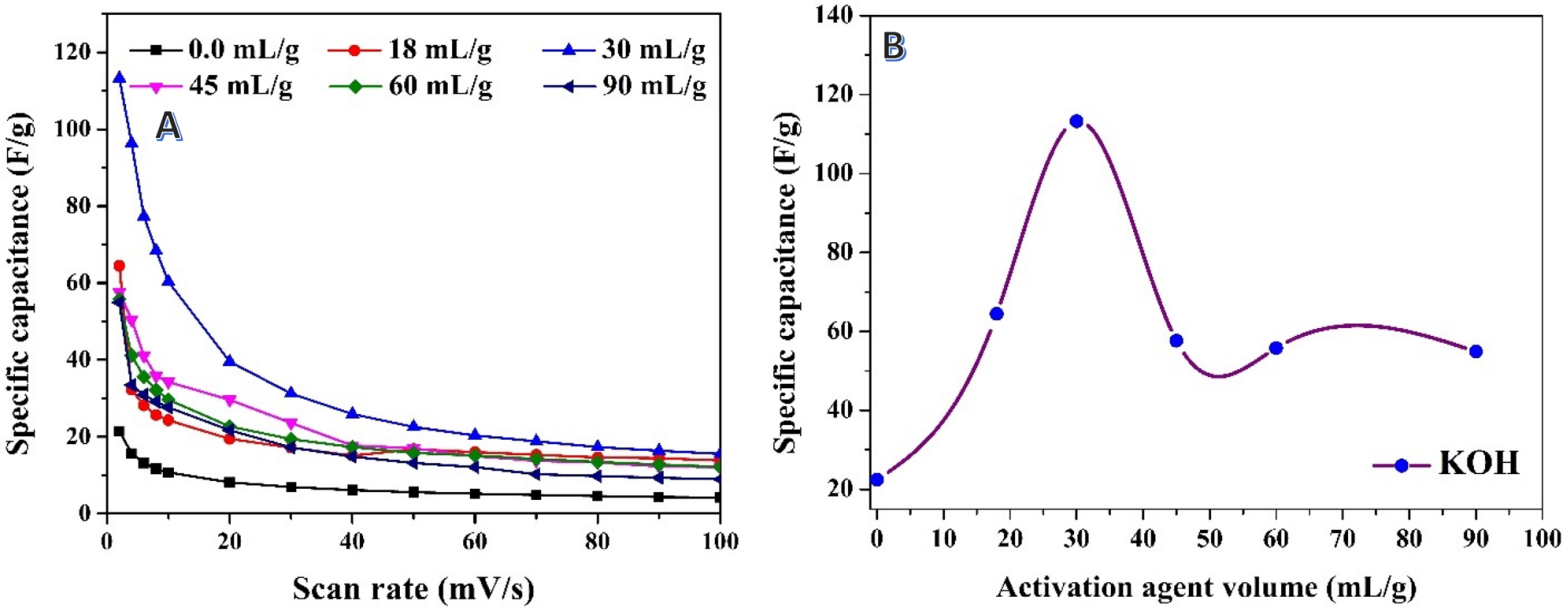
Relationships between scan rate and the specific capacitance at different volumes of the used potassium hydroxide solution (1.0 M) in activation of previously graphitized rice husk at 600 °C; (**A**), and effect of the used alkali volume on the specific capacitance at 2 mV/s scan rate; (**B**).

As discussed earlier, the specific capacitance is inversely proportional to the scan rate, as shown in Fig. 7A. The increase in scan rate restricts the accessibility of ions to the electrode surface. This phenomenon can be attributed to the ion sieving effect, where the ultrafine pores in the treated activated carbon can only be accessed at a slower diffusion rate^69,70^. Compared to H_3_PO_4_-activated carbon using the same route, which demonstrates the highest specific capacitance value of 73.5 F/g using a 21 mL/g solution, the utilization of KOH as an activation agent yields a higher maximum specific capacitance. As depicted in Fig. 7B, the highest specific capacitance value of 113.24 F/g is obtained when using a 30 mL/g volume of the alkali solution. However, further increase in the volume of the activation agent solution has a negative impact on the specific capacitance.

#### Prior-to-carbonization alkali treatment

**Figure 8:**
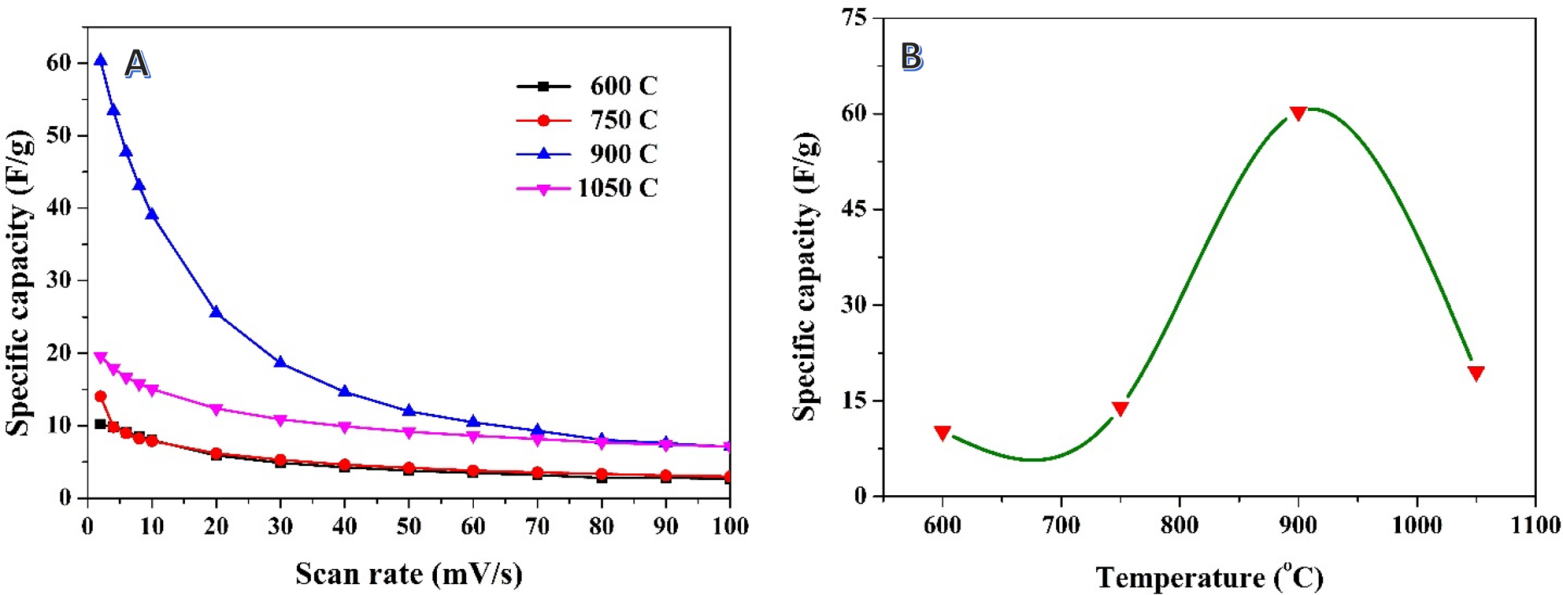
Relationship between scan rate and the specific capacitance at different carbonization temperatures for a previously soaked rice husk in potassium hydroxide solution (1.0 M) (**A**), and effect of the carbonization temperature on the specific capacitance at 2 mV/s scan rate (**B**).

The investigation of the impact of carbonization temperature on the activated carbon produced through alkali treatment before the carbonization process provides valuable insights into the specific capacitance behavior. Like the findings from the post-carbonization chemical treatment samples, the results (Fig. 8A) indicate a consistent trend where the specific capacitance decreases as the scan rate increases, regardless of the carbonization temperature. This observation is in line with the expected behavior, as higher scan rates impose limitations on the charge/discharge time and restrict ion diffusion, resulting in decreased specific capacitance values.

Interestingly, the investigation also reveals that the carbonization temperature significantly affects the specific capacitance of the activated carbon. Among the studied temperatures, the best carbonization temperature for achieving high specific capacitance is found to be 900 °C. At this temperature, as seen in Fig. 8B, the estimated specific capacitance reaches 60.3 F/g at a scan rate of 2 mV/s, representing a 95% decrease compared to the first route (referring to the alkali treatment after carbonization). The observed decrease in specific capacitance at higher carbonization temperatures suggests that excessively high temperatures may have a detrimental effect on the structural and electrochemical properties of the activated carbon. It is possible that higher temperatures lead to the formation of a more graphitic structure, which may not be as conducive to efficient charge storage as the structure formed at the optimal carbonization temperature.

These findings highlight the critical role of optimizing the carbonization temperature in determining the specific capacitance of the activated carbon when alkali treatment is performed before carbonization. It emphasizes the importance of carefully controlling the carbonization conditions to achieve the desired electrochemical performance in supercapacitors. Further investigations can delve into the underlying factors and mechanisms contributing to the observed decrease in specific capacitance at higher carbonization temperatures, providing valuable insights for the optimization of supercapacitor materials and performance.

### Electrode stability

The investigation of the stability of the two different routes, namely acid treatment prior to carbonization and alkali treatment after carbonization, provides valuable insights into the long-term performance and durability of the activated carbon for supercapacitor applications.

**Figure 9:**
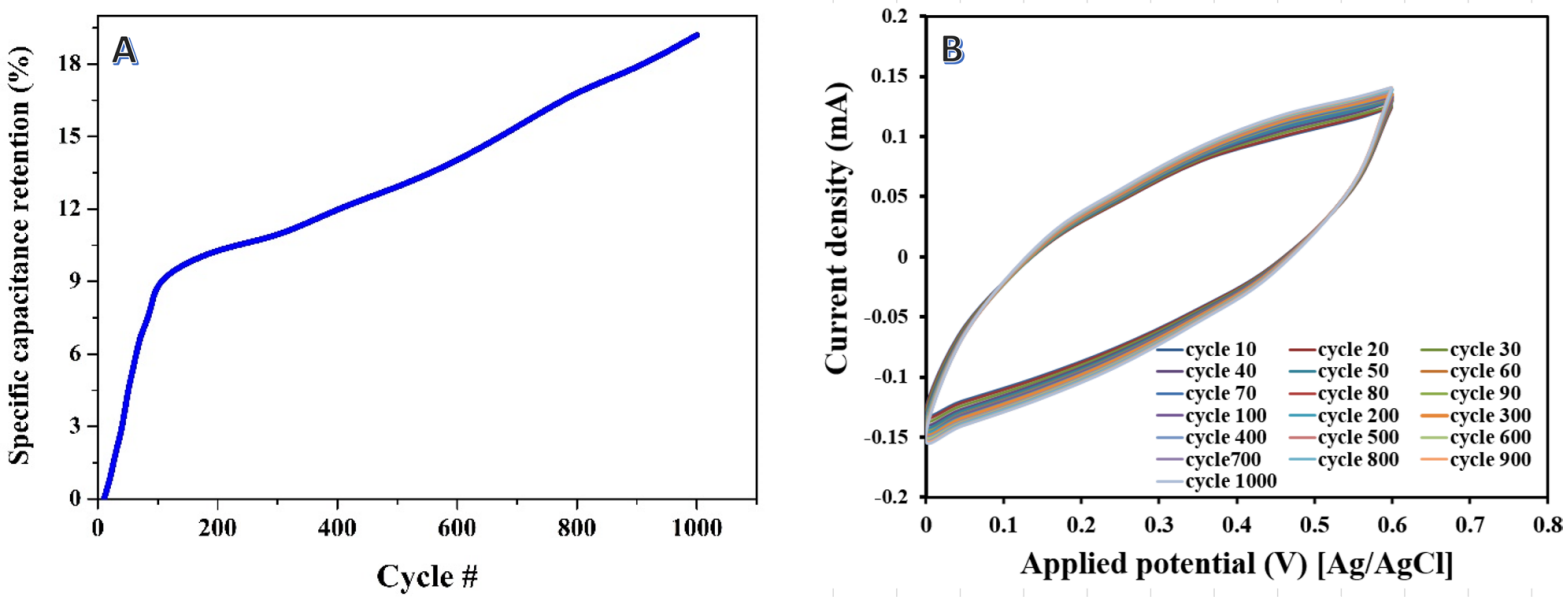
Variation of the specific capacitance (at scan rate of 5 mV/s) of the prepared activated carbon by soaking the rice husk in phosphoric acid and then graphitized at 900 °C (**A**), and cyclic voltammograms for some selected cycles (**B**).

In the case of acid treatment prior to carbonization, the results demonstrate a remarkable improvement in the specific capacitance over successive cycles as shown in Fig. 9A. As examples, the cyclic voltammograms of some selected cycles were plotted in Fig. 9B. The specific capacitance shows a continuous increase throughout the 1000 cycles, ultimately reaching an 18% increase compared to the initial specific capacitance. This suggests that the acid treatment route enhances the stability and performance of the activated carbon, allowing it to maintain and even improve its specific capacitance over prolonged cycling. As indicated by Elmouwahidi et al.^62^ that H_3_PO_4_-activated samples showed good stability, around 70% of retention capacitance, indicating the stability of the capacitance of the activated carbons does not depend only on the micropores structure but also on an adequate mesopore network, which favors the exchange of the ions.

**Figure 10:**
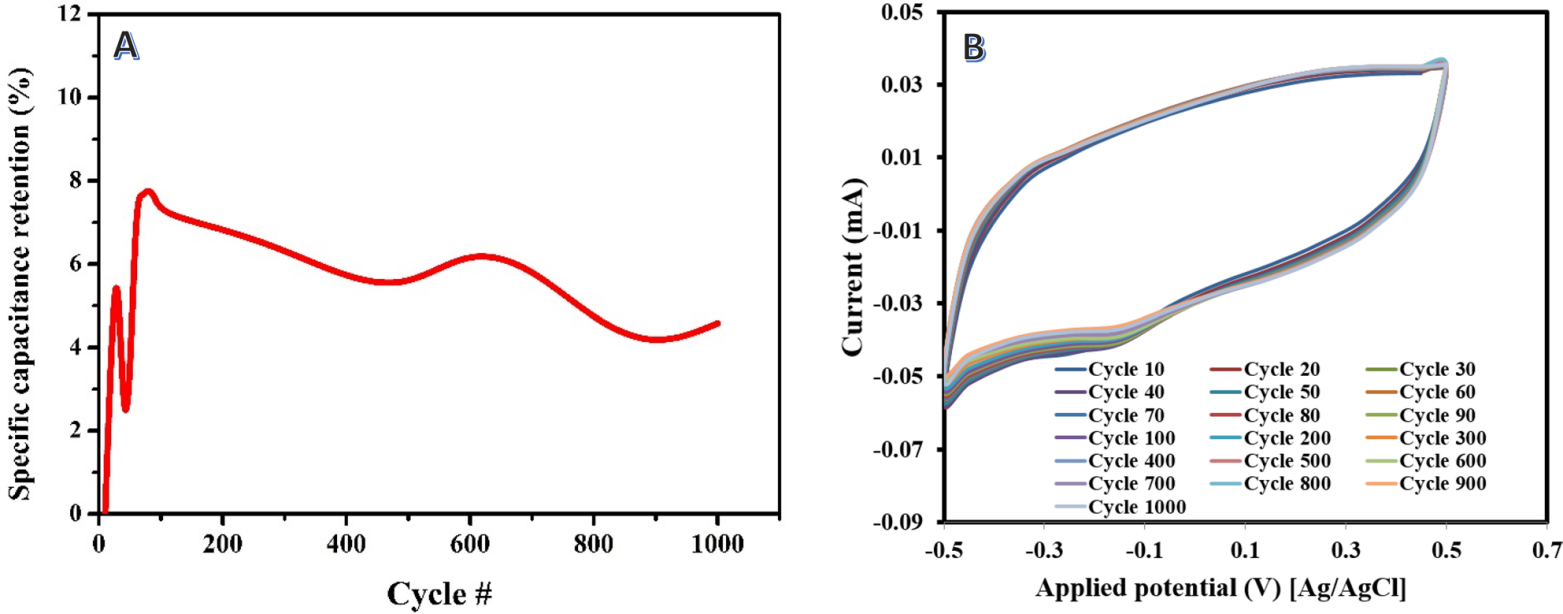
Variation of the specific capacitance (at scan rate of 5 mV/s) of the prepared activated carbon carbonization the rice husk at 600 °C and then activated by 45 mL/g 1.0 M KOH solution (**A**), and cyclic voltammograms for some selected cycles (**B**).

On the other hand, for the alkali treatment after carbonization route, as shown in Fig. 10A, a different trend is observed. The specific capacitance shows a slight decrease after the 100th cycle. However, within running 1000 successive cycles, the voltammograms are overlaid as shown in Fig. 10B. This indicates that the alkali treatment route may exhibit a relatively lower stability and durability over prolonged cycling. The decrease in specific capacitance suggests that the performance of the activated carbon may gradually deteriorate, possibly due to structural changes or degradation of active sites during cycling. The contrasting stability behaviors between the acid treatment and alkali treatment routes can be attributed to their different effects on the structural and surface properties of the activated carbon. The acid treatment route prior to carbonization may promote the development of a more stable and optimized carbon structure, leading to improved cycling stability and enhanced specific capacitance. On the other hand, the alkali treatment after carbonization route may introduce certain factors that contribute to performance degradation and decreased stability over time.

These findings highlight the importance of carefully selecting the treatment route and understanding its influence on the stability and long-term performance of the activated carbon for supercapacitor applications. Further investigations can delve into the underlying factors and mechanisms behind the stability behaviors observed in each route, aiding in the development of strategies to enhance the stability and durability of activated carbon for efficient and reliable supercapacitor applications. It is worth mentioning that checking the stability for more cycles (10,000 cycles) indicated almost similar conclusions about the stability for the prepared activated carbons as shown in Fig. S4 in the supporting information.

## Conclusions

In conclusion, this manuscript investigated the influence of the chemical activation step order and the carbonization temperature on the specific capacitance of activated carbon derived from rice husk. The findings provide valuable insights into optimizing the preparation process for enhanced supercapacitor performance. The results revealed that the specific capacitance of the activated carbon decreased with increasing scan rate, indicating a trade-off between specific capacitance and charge/discharge rate. When the chemical activation step was performed before the carbonization process, the best carbonization temperature was found to be 900 °C. At this temperature, the activated carbon exhibited a specific capacitance of 88.5 F/g at a scan rate of 2 mV/s, representing an 21% increase compared to the alternative route. Similarly, when the chemical activation step was performed after the carbonization process, the best carbonization temperature was also determined to be 900 °C. However, the specific capacitance obtained in this case was lower, with a value of 60.3 F/g at a scan rate of 2 mV/s. This indicated a 95% decrease in specific capacitance compared to the first route. Furthermore, the stability analysis demonstrated that the acid treatment prior to carbonization led to an improvement in specific capacitance over successive cycles, showing an 18% increase after 1000 cycles. In contrast, the alkali treatment after carbonization resulted in a slight decrease in specific capacitance after the 100th cycle, suggesting a relatively lower stability over long-term cycling. These findings highlight the significance of the chemical activation step order and the carbonization temperature and the volume of the activating agent in determining the specific capacitance and stability of the resulting activated carbon. By carefully selecting the appropriate preparation conditions, it is possible to optimize the performance of activated carbon for supercapacitor applications. Overall, this study contributes to the understanding of the factors influencing the specific capacitance and stability of activated carbon derived from rice husk. The findings provide valuable guidance for the design and optimization of preparation processes for high-performance supercapacitors, with potential applications in energy storage and other related fields.

### Supplementary Information

Supplementary Figures.

## Data Availability

All data generated or analyzed during this study are included in this published article.
