# Supplementary material for: Comparing specific capacitance in rice husk-derived activated carbon through phosphoric acid and potassium hydroxide activation order variations
Source: Sci Rep. 2024 Jan 17;14:1460. doi: 10.1038/s41598-023-49675-0 (PMC10794207; doi:10.1038/s41598-023-49675-0)
Supplement: Supplementary file 1 — Supplementary Figures. [file 41598_2023_49675_MOESM1_ESM.docx]

**Comparing Specific Capacitance in Rice Husk-derived Activated Carbon Through Phosphoric Acid and Potassium Hydroxide Activation Order Variations**

**Nasser A. M. Barakat^a,*^, Mohamed S. Mahmoud^a,b^ and Hager M. Moustafa^a^**

^a^ Faculty of Engineering, Chemical engineering department, Minia University, El-Minia, 61516, Egypt

^b^ Department of Engineering, University of Technology and Applied Sciences, Suhar, 311, Oman

**Corresponding author:**

Nasser A M Barakat

Tel: 0020862345008

Fax: 0020862346674

E-mail: [nasbarakat@mu.edu.eg](mailto:nasbarakat@mu.edu.eg)


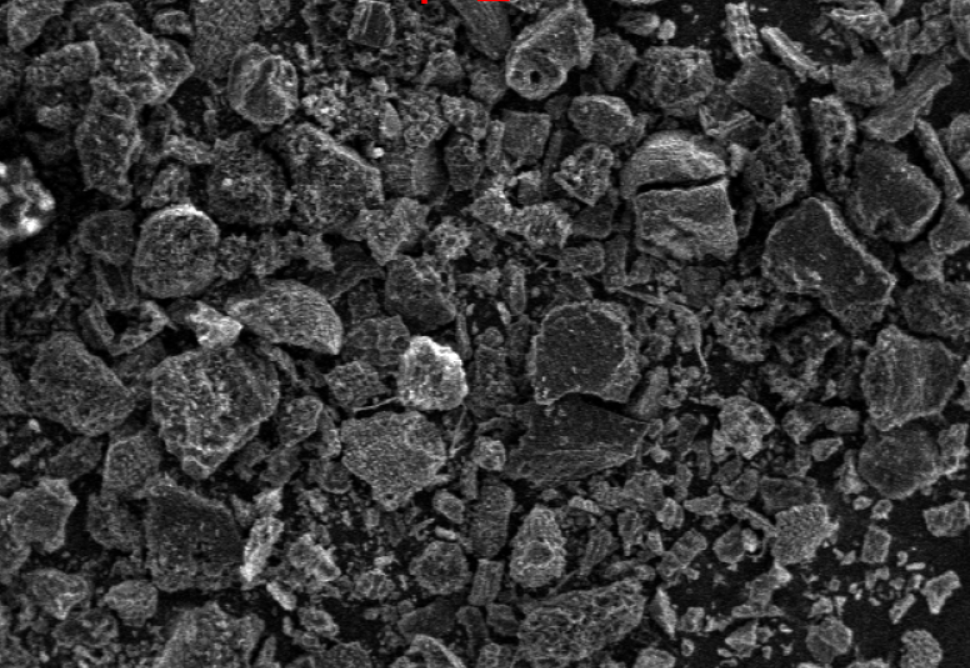

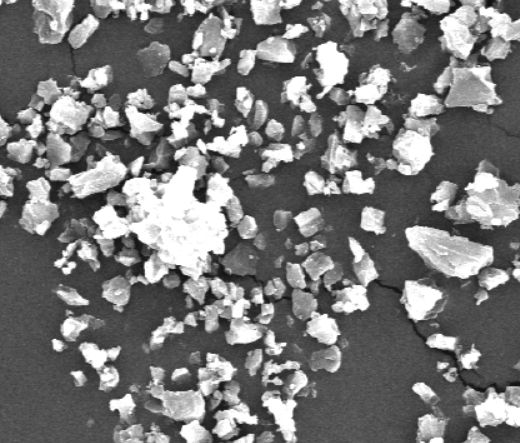


**Fig. S1 SEM images of the produced activated carbon using acid and alkali treatment, left and right, respectively.**

**
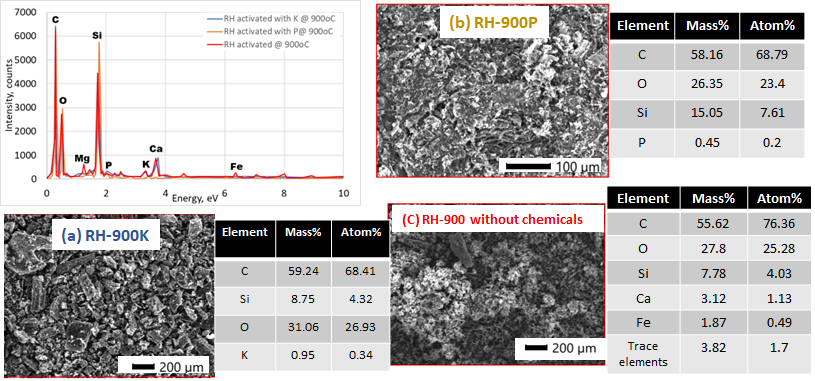
**

**Fig. S2 EDS and elemental analysis of rice husk activated: (a) by K solutions; (b) by P solution; and (c) pristine carbon obtained from of the rice husk carbonation @ 900 ^o^C.**

**Fig. S3 Cyclic voltammetry measurements for some prepared activated carbons.**

**Fig. S4** **Cyclic performance, stability, and capacity retention test of RH-900P at 2mV/s for 10000 cycles.**
